# Lineage selection and plasticity in the intestinal crypt

**DOI:** 10.1016/j.ceb.2014.07.002

**Published:** 2014-12

**Authors:** Anna Philpott, Douglas J Winton

**Affiliations:** 1 Department of Oncology, University of Cambridge, Hutchison/Medical Research Council (MRC) Research Centre, Cambridge CB2 0XZ, UK; 2 Cancer Research UK Cambridge Institute, University of Cambridge, Li Ka Shing Centre, Robinson Way, Cambridge CB2 0RE, UK

## Abstract

We know more about the repertoire of cellular behaviours that define the stem and progenitor cells maintaining the intestinal epithelium than any other renewing tissue. Highly dynamic and stochastic processes define cell renewal. Historically the commitment step in differentiation is viewed as a ratchet, irreversibly promoting a given fate and corresponding to a programme imposed at the point of cell division. However, the emerging view of intestinal self-renewal is one of plasticity in which a stem cell state is easily reacquired. The pathway mediators of lineage selection are largely known but how they interface within highly dynamic populations to promote different lineages and yet permit plasticity is not. Advances in understanding gene regulation in the nervous system suggest possible mechanisms.

## Highlights

• Cell fate choice in the crypt is controlled by bHLH transcription factors.
• Differentiated intestinal lineages display unexpected plasticity.
• Parallels with neurogenesis suggest oscillatory expression of bHLH proneural factors.
• Regaining oscillatory behaviour may be a perquisite for intestinal plasticity.

**Current Opinion in Cell Biology** 2014, **31**:39–45

This review comes from a themed issue on **Cell cycle, differentiation and disease**

Edited by **Stefano Piccolo** and **Eduard Batlle**

For a complete overview see the Issue and the Editorial

Available online 15th September 2014

**http://dx.doi.org/10.1016/j.ceb.2014.07.002**

0955-0674/© 2014 The Authors. Published by Elsevier Ltd. This is an open access article under the CC BY license (http://creativecommons.org/licenses/by/3.0/)

## Introduction

**Figure 1:**
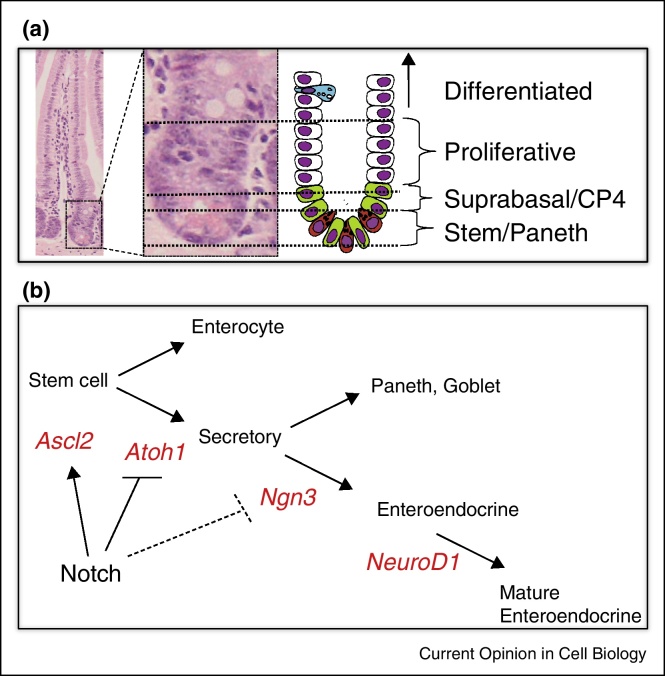
Organisation and lineage control in the intestine. **(a)** H&E section of intestine showing crypt-to-villus axis. Expanded view of crypt shown alongside a schematic showing the location of the different functional zones. (b) Schematic of classical view of bHLH transcription factor-driven control of fate choice and differentiation in the intestine, and a simplified view of their regulation by Notch signalling. However, complex interaction between cells, potential oscillating expression of bHLHs, and a clear ability to move back up the hierarchy towards stemness, points strongly to a great deal of potential for plasticity, rather than cells following a linear pathway as depicted here.

The sheet of cells that comprises the small intestinal epithelium is indented to create glandular crypts in which cell proliferation is restricted and from which all cell types are generated. Absorptive enterocytes and secretory (Goblet and enteroendocrine) cells actively migrate from crypts while undergoing a phenotypic maturation that is accompanied by a restricted number of transient cell divisions (Figure 1). The most morphologically undifferentiated cells are located at or near the crypt base where they interface with long-lived differentiated secretory Paneth cells. These undifferentiated cells are maintained by robust levels of active Wnt signalling, characterised by expression of Lgr5 (a R-spondin receptor) and contain much of, and arguably all, the steady-state stem cell activity as shown by lineage tracing. The colonic epithelium has similar organisation but lacks both villi and Paneth cells.

There are differences in the properties of cells in the crypt base which are recognised by heterogeneous expression of markers and that arises from both the geography of the lower crypt and the availability of Paneth cells for cell-cell interaction. Together these factors create a nuanced biology; undifferentiated cells immediately above the Paneth cell region (at, or around, cell position 4 from the crypt base) tend to express different markers than those within it. The cells within these different zones have been proposed as alternative candidates for the stem cell population. Position specific heterogeneity in marker expression and in properties such as quiescence has previously been interpreted as indicative of relatively stable subpopulations moving unidirectionally through discrete cellular intermediates from multipotent stem cells to committed progeny. However, recent evidence for plasticity challenges this interpretation and suggests that normal cell fates are easily altered and stemness regained.

## Intestinal lineage specification by Notch and the bHLH proteins

Historically attempts to explain how multiple phenotypically distinct cell types arise within the crypt have assumed the creation of lineage-restricted progenitors that can be distinguished by different transcription factor profiles [1, 2]. Commitment has been viewed as a series of binary decisions, the first directing absorptive versus a ‘pan’ secretory fate, followed by further diversification into the four principal secretory types [3].

Several key bHLH ‘proneural’ proteins play distinct and crucial roles in early lineage specifications as well as differentiation events in the crypt, and their expression and activity are spatially and temporally regulated (Figure 1). A large part of this regulation appears to be via the Notch signalling pathway [4, 5, 6, 7].

Ultimately Notch signalling regulates the stem versus secretory fate decision as well as further fate choice and differentiation events in the crypt [8, 9]. Expression of the proneural bHLH transcription factor Ascl2 is associated with stemness and is absolutely required for intestinal stem cell maintenance. Active Notch is required for Ascl2 expression and its loss results in precocious crypt cell differentiation [8, 10]. The proneural protein Atoh1 acts as a master regulator of fate specification of the secretory lineage [2, 11]. Ascl2 expression is maintained by active Notch signalling that also acts to suppress Atoh1. Expression of Atoh1 is cell-autonomously inhibited by Hes proteins and in the absence of Notch signalling, crypt stem cells precociously differentiate into secretory goblet cells [7, 12].

**Figure 2:**
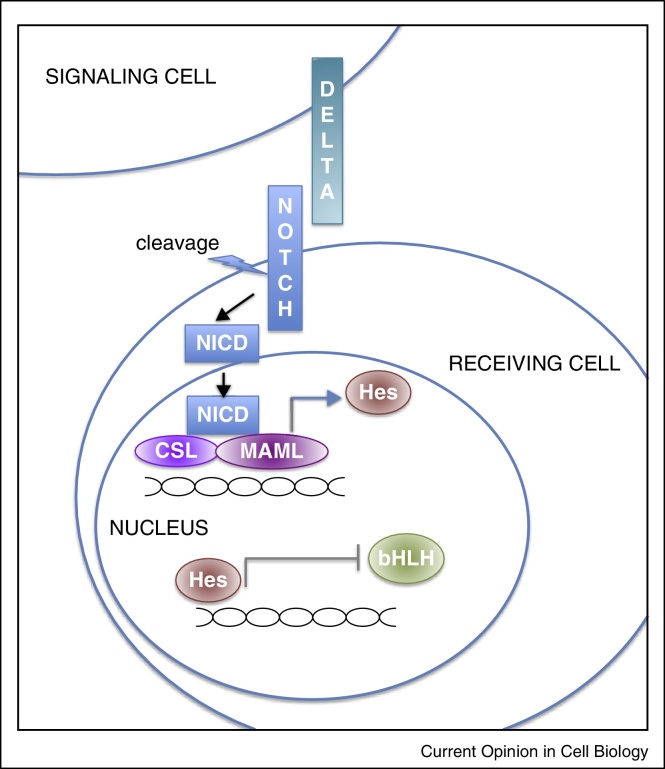
Schematic of the Notch signalling pathway. In brief, activation of the Notch membrane receptor requires binding by a member of the membrane-bound ligand Delta family (primarily Delta-like, Dll 1 and 4 and Jag 1 in the crypt) [9]. Binding of ligand to the receptor leads to release of the Notch intracellular domain (ICD) by protein cleavage. NICD translocates to the nucleus and associates with the CSL complex (CBF-1/RBP-J, Su(H), Lag1), displacing transcriptional repressors. This complex now associates with transcriptional co-regulators of the MAML family, resulting in upregulation of multiple downstream targets including Hes (Hairy/Enhancer of Split) proteins. Notch signalling via Hes proteins act to potentiate stem cell maintenance and inhibit secretory via regulation of bHLH transcription factors. For many more details see [5].

The spatial organisation of cells expressing Notch ligand and receptor in the crypt evokes a classic lateral inhibition scenario for control of stem versus secretory fate (Figure 2). Stem cells towards the crypt base found preferentially adjacent to Delta-expressing Paneth cells, express Notch receptor [13•, 14], and are maintained in an undifferentiated state by constant Notch signalling and suppression of Atoh1 [7, 9, 15, 16], As migrating cells lose contact with Paneth cells and the high Notch signalling they confer, they become poised between secretory and non-secretory fate. Lineage selection may then arise by stochastic variation in Delta expression leading some cells to express higher levels than others. This initial stochastic imbalance in Delta expression becomes reinforced allowing only a subset of cells (Delta high, Atoh high) rising up the crypt to become committed to a secretory fate while the rest become absorptive enterocytes.

This regulation and functional organisation readily explains a binary fate in a supra-Paneth cell poised population but fits less well with a subsequent downstream cascade of secretory lineage choices specified after a series of cell divisions each progressing unidirectionally towards a more restricted fate. Moreover, recent evidence derived from regenerating systems casts doubt both on the existence of stable populations of progenitors and the irreversibility of lineage specification.

## Plasticity

**Figure 3:**
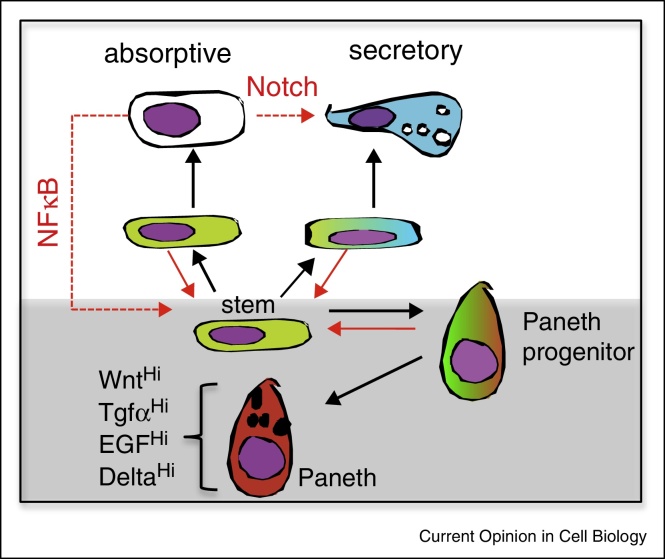
Schematic showing routes for stem cell restoration. Normal differentiation (black arrows) follows unidirectional lineage choice via intermediates. Stem cells occupy a sustaining environment created by Paneth cells (grey box). Following damage regenerative processes allow stemness to be regained (solid red arrows) from immediate stem cell descendants. Experimental upregulation of pathways shown can act to effect lineage fates from differentiated cells (dashed arrows).

For many years it has also been known that intestinal regeneration following damage is not solely a function of surviving stem cells expanding to restore homeostasis (Figure 3) [17]. Following radiation induced injury the clonogenic fraction of crypt cells is elevated suggesting that these might correspond to the abundant and immature absorptive cells present within the early transit-amplifying compartment of the lower crypt. In support, specific ablation of the key Lgr5+ population using targeted diptheria toxin is not catastrophic as non-Lgr5+ cells (Bmi1+) cells are able to act as a replacement stem cell pool at least for a limited time [18]. Strikingly though, Lgr5+ cells do appear to be essential for intestinal regeneration after irradiation, indicating that context of either the initial damage and/or the subsequent regenerative response may reveal plasticity in different populations [19^•^]. Even in steady state conditions, some interconversion occurs between Lgr5+ cells and cells residing at higher crypt levels, defined by Hopx expression indicating a ready accessibility of early committed cells to the stem compartment [20].

Recent discoveries indicate more dramatic plasticity within the absorptive lineage (Figure 3). Hyperactivation of pathways synergising with Wnt signalling are apparently able to generate stem cells as part of an oncogenic process even within terminally differentiated villus cells [21^••^]. Hyper-elevation of NF-κB signalling, by deletion of negative regulators of the pathway, synergises with Wnt signalling, elevating targets such as Ascl2 and leading to ectopic formation in villi of crypt-like structures expressing stem cell markers [21••, 22]. Further 3-D spheroid culture of isolated villi confirms the potential of these cells to proliferate over several passages and show multilineage differentiation in xenografts.

Evidence that secretory progenitors can also contribute to regeneration comes from functional studies of cells expressing Delta-like 1 (see below). Lineage tracing in Dll1-CreER mice following Tamoxifen treatment demonstrates that single Dll1+ cells in the steady state give rise mainly to short lived secretory clones [13^•^]. Equivalent lineage tracing following damage shows that many Dll1+ cells can give rise to long lived clones comprising both absorptive and secretory lineages, demonstrating that they have regained stem cell activity [13^•^]. Further, elevated Notch signalling in intestinal villi can cause phenotypic switching of mature differentiated cells from an absorptive to secretory lineage [23].

Subsequently the status of quiescent or label-retaining cells (LRCs) in the epithelium was investigated using a conditionally expressed, histone-conjugated fluorescent protein (H2BYFP) that could be widely induced initially and subsequently retained in cells that are quiescent [24^••^]. Characterisation of isolated YFP-LRCs shows these cells have a secretory signature associated with Paneth and enteroendocrine cells. Moreover, inheritance of the label into these cell types is observed over time. Functional lineage tracing of these YFP-LRCs shows that they do not normally give rise to multilineage clones but do so after regenerative stimuli. Together these findings suggest that quiescent cells are committed to become Paneth and enteroendocrine cells but after damage and regeneration are capable of reacquiring stem cell potential.

In summary both absorptive and secretory lineages display plasticity in experimental settings. For cells of either type, plasticity requires responsive cells not only to proliferate but also to demonstrate acquisition of the opposing phenotype, that is, multipotentiality.

## Notch and bHLH proteins regulate cell fate and plasticity

The classical model of Notch-mediated lateral inhibition, whereby initially equivalent cells interact with each other to adopt alternative fates, was originally formulated to describe the specification of individual neural precursors from an equivalence group of cells under the control of a network of bHLH proneural transcription factors and Hes proteins, analogous to those in the gut [25]. Yet this model notably fails to explain intestinal plasticity where the reverse applies, that is, the acquisition of stem cell ‘equivalence’ from phenotypically diverse cells. Again, advances in our understanding of mammalian neurogenesis indicate the potential for a more dynamic regulation of these types of specification events than originally proposed that may help explain intestinal plasticity.

**Figure 4:**
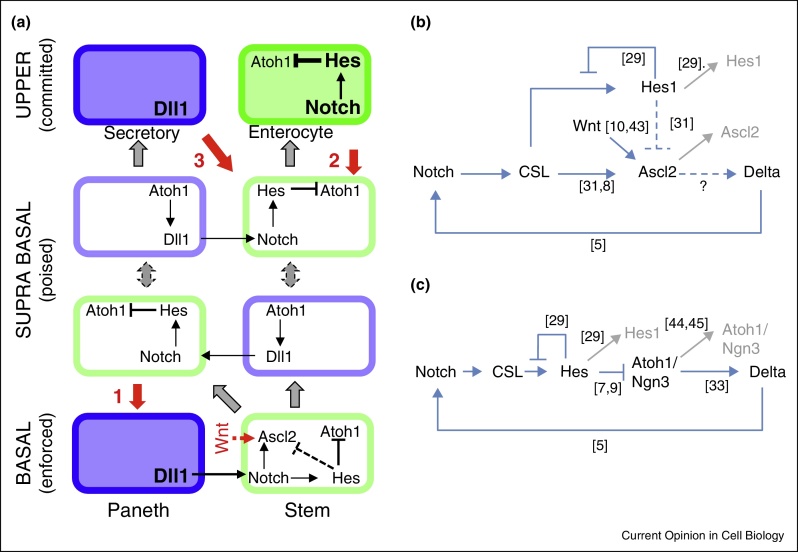
Potential interaction nodes for intestinal lineage specification. **(a)** In the crypt base cells are maintained in a WntHi environment in which high Notch signalling in stem cells is sustained by long lived secretory cells expressing Delta-like ligands. In suprabasal crypt positions oscillatory expression allows cells to move between lineage poised fates (secretory and absorptive). This resolves stochastically and at higher crypt positions cells are lineage committed. Stem potentiality following damage/regeneration by: **(1)** restored interactions with Dll1 expressing cells; **(2)** by hyper stimulation of key pathways such as Wnt/NFkB in committed cells. **(3)** In experimental/extreme settings mature cells expressing Delta ligand may promote local re-initiation of Notch signalling **(b,c)**. Potential molecular circuitry linking Notch signalling and bHLH transcription factor expression for Ascl2 (B) and Atoh1/Ngn3 (C), based on known direct and indirect interactions [5, 7, 8, 9, 10, 29, 31, 33••, 43, 44, 45]. Solid blue arrows denote relationships supported by evidence in the intestine. Dotted blue lines indicate relationships inferred from the same gene in other tissues or by analogy to closely related bHLH transcription factors. Grey arrows and text indicate protein instability; where studied, these bHLHs and Hes proteins have been shown to have short, and sometimes exceptionally short, half lives. The delayed negative feedback loop in (B) has been shown to result in oscillatory expression of analogous Hes and bHLH proteins in neural stem cells [29].

In the mammalian nervous system, expression of the proneural bHLH transcription factors Ngn2 and Ascl1 oscillates with a periodicity of 2–3 hours in neural stem/progenitor cells. Oscillations are controlled by a transcriptional double negative feedback loop; the proneural transcription factors control expression of Delta-like ligands, activating Notch signalling and consequently resulting in delayed anti-phased expression of short-lived repressors (the Hes proteins) [26, 27••]. Such Notch/Delta-mediated interactions between adjacent cells result in reciprocal Delta, bHLH and Hes oscillations where neighbours are out of synchrony and progenitor maintenance prevails [27••, 28]. Cessation of oscillations of both proneural and Hes proteins coincides with fate choice decisions, and results in sustained high expression of proneural proteins to drive differentiation, with reciprocal sustained low expression of Hes inhibitors. Indeed, in the nervous system stable, as opposed to oscillatory, bHLH expression seems to be absolutely required for cells to exit the cell cycle and adopt a differentiated fate [27••, 28, 29]. As the essential players in fate decisions in the crypt are highly analogous to those in the nervous system, it seems likely that such oscillatory expression of proneural and Hes proteins also occurs in the intestine. For instance, Atoh1 upregulates Delta expression and is itself repressed by Notch and Hes activity [5, 9], so is well-placed to be part of a similar double negative feedback loop driving oscillatory expression as is seen for Hes1, Ngn2 and Ascl1 (Figure 4) [29, 30]. Active Notch is required for Ascl2 expression but may also have contradictory effects as Hes1 has been described as suppressing Ascl2's expression in epidermal cells [31]. Ascl2 can also be directly activated by Wnt and has a crucial role in maintaining stemness [8, 10, 31]. Speculatively, oscillatory expression of Ascl2 may be required for this function, as is the case for Ascl1 and neural stem cell maintenance.

Where in the crypt stem/progenitor pool might such oscillations operate? This will be hard to determine *in vivo* with current methodologies, as all oscillatory expression will probably fall beneath the detection threshold of common visualisation techniques [26, 27••]. There may be clues however from studies of Dll1 where *in situ* hybridisation indicates that high (and maybe stable) Delta expression occurs in supra-Paneth cell positions in cells that also express high levels of Atoh1 (Figure 4) [13^•^]. Low-level oscillations may occur at the lower cell positions containing the intercalated, Lgr5+ population. Additionally, lower levels of Delta are seen in individual cells higher in the crypt and even on the villus (though the bHLH and Hes proteins are not), commensurate with Notch signalling playing roles later in the specification/differentiation programme (see below) [13^•^].

Notch also regulates Ngn3, a bHLH that is absolutely required for secretory cells to adopt enteroendocrine fate [32]. The molecular mechanism of regulation of Ngn3 by Notch signalling is analogous to the regulation of Atoh1 as well as Ngn2 in the nervous system; where Notch activation inhibits Ngn3 expression, suppressing enteroendocrine cell formation and promoting alternate enterocyte or goblet fates [7, 33••, 34]. It is striking that enteroendocrine numbers are limited but not eliminated by Notch activation in Ngn3 positive cells while Notch activation driven by the villin promoter, that acts earlier in crypt specification results in complete enteroendocrine cell loss showing context-dependence of Notch sensitivity [33••, 35].

## Concluding remarks

In terms of plasticity the iterative role of Notch signalling means that the pathway is accessible to cells throughout the crypt to villus axis. After epithelial cell depletion, surviving cells have a number of options to be restored to a stem cell state. At the level of an individual cell this may require regaining low-level oscillatory Notch signals associated with the poised state perhaps by altering the stability or post-translational regulation of the bHLH proteins that promote fate decisions [36]. Alternatively, in maturing enterocytes [37, 38], upregulation of Hes family proteins could actively promote Ascl2 while suppressing Atoh1 expression and function. Notably the Ascl2 axis with potentially competing roles for elements of the Notch pathway also allows input and crosstalk from the Wnt pathway. Cell interactions favouring acquisition of stemness might include occupying a vacant cell position adjacent to a DeltaHi expressing cell to promote active Notch signalling in neighbours.

The outline circuitry defined by the bHLH/Hes axis regulation can be fleshed out by a variety of post-transcriptional interactions and modification to limit or potentiate available Notch signalling in a context dependent manner. For example Notch transcript itself can be sequestered by regulatory microRNAs such as miR-34a. Downregulation of miR-34a following damage could promote not only acquisition of stemness but allow for rapid expansion of stem cells by symmetric divisions [39^•^]. Post-translational interactions such as Numb-mediated degradation of membrane-bound Notch or translational inhibition of Numb by RNA binding proteins such as Musashi1 could similarly act to inhibit or promote Notch signalling respectively [40, 41].

Finally, recently it has been shown that the chromatin status cells of secretory and absorptive progenitors remain constant. It is likely that throughout the crypt the palette of accessible loci remains unchanged with lineage choice making the restoration of stemness from maturing cell types purely dependent on expression on key transcription factors [42^••^]. In confirming the dependency of the epithelium on bHLH family members attention must turn to determining their modes of expression and how these are regulated to achieve different outcomes in different contexts including both in homeostasis and the plasticity associated with regeneration.

## References and recommended reading

Papers of particular interest, published within the period of review, have been highlighted as:

• of special interest
•• of outstanding interest
